# A versatile high-throughput assay to characterize antibody-mediated neutrophil phagocytosis

**DOI:** 10.1016/j.jim.2019.05.006

**Published:** 2019-08

**Authors:** Christina B. Karsten, Nickita Mehta, Sally A. Shin, Thomas J. Diefenbach, Matthew D. Slein, Wiktor Karpinski, Edward B. Irvine, Thomas Broge, Todd J. Suscovich, Galit Alter

**Affiliations:** a Ragon Institute of MGH, MIT and Harvard, 400 Technology Square, Cambridge, MA 02139, USA; b Harvard T.H. Chan School of Public Health, 677 Huntington Ave, Boston, MA 02115, USA

**Keywords:** Non-neutralizing antibody, Antibody-dependent neutrophil phagocytosis, Systems serology, High-throughput assay, Immune correlates of protection

## Abstract

Neutrophils, the most abundant white blood cell, play a critical role in anti-pathogen immunity via phagocytic clearance, secretion of enzymes and immunomodulators, and the release of extracellular traps. Neutrophils non-specifically sense infection through an array of innate immune receptors and inflammatory sensors, but are also able to respond in a pathogen/antigen-specific manner when leveraged by antibodies via Fc-receptors. Among neutrophil functions, antibody-dependent neutrophil phagocytosis (ADNP) results in antibody-mediated opsonization, enabling neutrophils to sense and respond to infection in a pathogen-appropriate manner. Here, we describe a high-throughput flow cytometric approach to effectively visualize and quantify ADNP and its downstream consequences. The assay is easily adaptable, supporting both the use of purified neutrophils or white blood cells, the use of purified Ig or serum, and the broad utility of any target antigen. Thus, this ADNP assay represents a high-throughput platform for the in-depth characterization of neutrophil function.

## Highlights

• A high-throughput antibody-dependent neutrophil phagocytosis (ADNP) assay was developed.
• This flow cytometry assay is flexible and can be easily adapted to any pathogen.
• Analysis of sample sets by ADNP assay is fast, robust and cost-effective.
• Additional neutrophil functions can be profiled in secondary analyses.

## Graphical abstract

**Figure:**
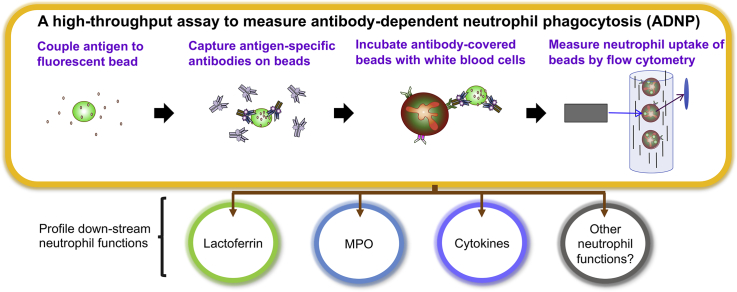

## 1 Introduction

Neutrophils make up 40–70% of circulating leukocytes in the adult population (Infection, C. f. D. C. a, 2017) and are the first responders following a wide range of infections including bacteria (Brinkmann et al., 2004; Sanders et al., 1995), fungi (Gazendam et al., 2016), as well as viruses (Galani and Andreakos, 2015; Naumenko et al., 2018; Orenstein, 2000; Saitoh et al., 2012). Although short lived, they are abundant, and their ability to be recruited to the site of infection render them key players in the immune system. Neutrophils kill pathogens and confer protection via a combination of mechanisms. Following pattern recognition receptor-mediated activation, neutrophils can take up foreign material, release reactive oxygen species, secrete antimicrobial proteases, and release their DNA to form neutrophil extracellular traps (NETs) (Barr et al., 2018; Aleyd et al., 2014; Williams, 2006).

Beyond pattern-based activating signals, neutrophils also express high levels of Fc receptors (FcR) and complement receptors (CR) (Akerley 3rd et al., 1991; Worley et al., 2018), enabling them to respond, clear, and destroy antibody-opsonized targets. Antibody mediated FcR and/or CR activation often results in antibody-opsonized immune complex uptake in a process termed antibody-dependent neutrophil phagocytosis (ADNP) that then enables the neutrophil to degrade, sense the cargo, and the deploy a pathogen-appropriate response.

Recent data suggest that beyond pathogen cargo, the quality of the antibodies themselves can prompt different neutrophil functions (Ackerman et al., 2018). Specifically, while variation in immune complexes drive pathogen clustering, variation in isotype and subclass composition as well as Fc-glycosylation can dramatically affect FcR and CR activation (Lux et al., 2013; Subedi and Barb, 2016; Davies et al., 2001; Nose and Wigzell, 1983) thereby altering pathogen-clearance mechanisms. Moreover, while neutrophils express constitutive levels of FcgR2a, FcgR3b, FcαR, CR1 (CD35) and CR3 (CD11b/CD18), upon activation, FcR/CR expression profiles change, further allowing neutrophils to change their responsiveness to antibody-opsonized targets (Mayadas et al., 2014; Berger et al., 1989). Thus, antibody Fc-mediated recruitment of neutrophils offers a unique opportunity to leverage the remarkable number and diverse biological functions of neutrophils via immunotherapeutics or vaccines. Given their critical biological role, wide biodistribution, and rapid ability to respond to infection or tissue injury, assays able to probe antibody-mediated neutrophil activation are crucial.

While several groups have made efforts in developing approaches to assess neutrophil functions including NETosis, degranulation and antibody-dependent respiratory burst (Gavillet et al., 2015; Kraaij et al., 2016; Llewellyn et al., 2015), a versatile high-throughput flexible-sized bead-based primary neutrophil ADNP assay for large-scale qualifiable direct analysis of clinical samples is still lacking. Many existing protocols measuring ADNP use fluorescent markers, pH-dependent dyes to label pathogens, or use antigen-expressing target cells (Grasse et al., 2018; Dri et al., 2002; Rodríguez et al., 2001; Richard et al., 2018) often reducing throughput. Additionally, the use of labeled bacteria or infected cells, while critical for immunogenicity or correlate analyses, provide little opportunity to resolve potential antigen-specific protective epitope targets. Moreover, in the context of HIV-infected cell-based assays, emerging data suggest inconsistent results may be captured using infected cells, due to the large number of conformations that may be adopted by the HIV viral envelope glycoprotein present on the infected cell, the infectious virus, or on virus-adsorbed cells, the latter exposing epitopes that are irrelevant in blocking infection, highlighting the need for assays able to present fixed conformations of target antigens (Richard et al., 2018). In contrast, we and others have shown that the use of fluorescent beads coated with defined antigens allow for the simultaneous investigation of antibody specificity and functionality (Worley et al., 2018; Ackerman et al., 2011). However, to date, bead-based assays adapted specifically for the investigation of neutrophil functions using primary human neutrophils for large scale cohort analysis are still lacking.

Here, we describe a flow cytometric-based method using target antigen-coupled fluorescent beads for the investigation of ADNP against virtually any target. This high-throughput assay enables the analysis of not only phagocytic uptake, but many secondary neutrophil functions that may contribute to and underlie antibody mode of action.

## 2 Materials and methods

### 2.1 Source of human samples

Primary human cells were isolated from blood of healthy donors collected by the Ragon Institute of MGH, MIT and Harvard. Serum samples from 2 healthy and 27 chronically infected human immunodeficiency virus 1 (HIV-1)^+^ patients were obtained from the Ragon Institute of MGH, MIT and Harvard as a source of pathogen-specific antibodies. All subjects provided informed written consent. The study was conducted in accordance with the World Medical Association's Declaration of Helsinki and approved by the MGH Institutional Review Board (approval# 2010P002463 and 2010P002121).

### 2.2 Isolation of cells

To generate white blood cells (WBC), red blood cells were lysed from whole human blood, which was collected using either ethylenediaminetetraacetic acid (EDTA) or acid citrate dextrose (ACD) as a coagulant. Blood was mixed at a 1:10 ratio with ammonium-chloride‑potassium (ACK) lysis buffer (150 mM NH_4_Cl, 10 mM KHCO_3_, 0.1 mM Na_2_EDTA, pH 7.4) and incubated for 5 min at room temperature. The WBCs were pelleted by centrifugation (500 ×*g*, 5 min) at room temperature and then washed with cold phosphate-buffered saline (PBS). For the isolation of human neutrophils, whole blood was mixed with hetasep (Stemcell) in a 1:5 ratio and incubated for 30 min to separate leukocytes from erythrocytes. Subsequently, the straw-colored layer of leukocytes was transferred to a fresh tube and neutrophils were isolated using the direct human neutrophil isolation kit (Stemcell) according to manufacturer's instructions. Both WBCs and purified neutrophils were finally diluted in complete R10 media (RPMI-1640 media (Sigma) with 10% FBS, 2 mM L-Gluthamine and 100 U/ml penicillin/streptomycin) for each assay.

### 2.3 Coupling of fluorescent beads

To generate antigen-coupled beads HIV-1 YU-2 gp120 (Duke Human Vaccine Institute), a mix of influenza HA1 proteins ((H1N1 A/California/07/2009; H3N2 A/Texas/50/2012; B/Massachusetts/2/2012), Immune Technology), Ebola virus Zaire GP (Immune Technology) or tetanus toxoid (MassBiologics) were biotinylated with Sulfo-NHS-LC biotin (Thermo Fisher) following the manufacturer's instructions. Unbound biotin was removed using a Zeba Spin desalting column (Thermo Fisher). The antigen was coupled in a 1:1 ratio to yellow-green fluorescent NeutrAvidin Fluospheres of 1 μm size (Invitrogen), which are stable across pH changes occurring during phagocytosis. Of note, beads of different sizes may be selected to mimick virus, bacterial, or cell size in immune complexes of interest. Additionally, antigen densities may further be adapted to probe for functionality in the setting of sparsity or variable density to accommodate user desired functional modeling. Specifically, beads were coupled to the biotinylated protein in low affinity tubes in the dark for 2 h at 37 °C or overnight at 4 °C. Subsequently, coupled beads were washed with 100× volume of PBS (16,000 ×g, 10 min) and resuspended in 100× volume of PBS with 0.1% BSA.

### 2.4 ADNP assay

For each ADNP assay, purified pooled Ig from HIV^+^ patients (HIVIG) (NIH) and Ig from HIV-negative (HIV^−^) patients (IVIG) (Sigma) were used as positive and negative controls, respectively. In a 96-well round bottom plate, 10 μl of diluted HIVIG, IVIG, sample or PBS alone, as a background control, were incubated with 10 μl of antigen-covered beads per well over the course of 30 min-2 h at 37 °C to allow immune-complex formation. Subsequently, the beads were washed in 200 μl of PBS, pelleted (10 min, 2000 ×g), and the supernatant was removed to clear out unbound Ig. WBCs or purified neutrophils were adjusted to a concentration of 2.5x10^5^cells/ml in R10, and 200 μl (50,000 cells/well) of cell suspension was added per well. The plate was incubated for 1-24 h at 37 °C in a humidified incubator. To collect the supernatant for downstream assays, the cells were pelleted (5 min, 4 °C, 500 ×g) and the supernatant was stored at -20 °C for future analysis. The cells were stained with CD66b-PacBlue alone (Biolegend) or additionally with fixable live/dead-near infrared stain (Invitrogen) and Annexin V-PE (Biolegend) and fixed with 4% paraformaldehyde. The assays were measured with a BD LSR2 or BD LSR Fortessa flow cytometer. Neutrophil bead internalization was quantified using FlowJo (FlowJo, LLC) software by gating for granulocytes (FSC/SSC)/neutrophils (CD66b^+^)/bead^+^neutrophils (FITC^+^). A minimum of 2000 cells were acquired and a phagocytic-score (phagoscore) was calculated for each sample comprising of the percent neutrophils that had taken up beads multiplied by the fluorescent signal of beads taken up by the neutrophils (geometric mean fluorescence intensity (gMFI) of (bead+ neutrophils)).

$$\mathrm{Phagoscore}=\frac{\text{gMFI}\left({\text{bead}}^{+}\;\text{neutrophils}\right)\times \left(\%{\text{bead}}^{+}\;\text{neutrophils of total neutrophils}\right)}{10,000}$$

In addition to the calculation of the phagoscore at a given sample concentration, an area under the curve (AUC) for phagoscores can be captured at different sample diutions using Prism 7 (Graphpad) software to detect differences in the phagocytic ability of samples at a higher resolution.

To define the specific Fc-receptor involved in driving ADNP, Fcγ-receptor blockade was performed using using HIVIG and IVIG and HIV-1 YU-2 gp120 as a target protein. The WBCs for this assay were pre-treated at a cell density of 5 × 10^5^/ml for 1 h at 4 °C using 5 μg/ml of either anti-FcγRI (Thermo Fisher), II (Bio X cell), III (Biolegend) or all anti-FcγRs in combination.

### 2.5 Antibody titers

A luminex assay was used to determine relative IgG titers to the HIV-1 YU2 gp120 antigen, as described previously (Brown et al., 2012). Briefly, magnetic carboxylated fluorescent beads (Luminex Corporation) were coupled to gp120 in a two-step carbodiimide reaction. Serum samples diluted at 1:100 were then incubated with antigen-coated beads overnight at 4 °C. Following bead washing, gp120-specific IgG1 was then detected with PE-conjugated mouse anti-human IgG1 (Southern Biotech #9052-09), diluted to 1.3 μg/ml in assay buffer, to each well. Median Fluorescence Intensity corresponding to each sample was measured using a BioPlex array reader (BioPlex 3D, Bio-Rad). A minimum of 50 beads per well were collected for analysis.

### 2.6 ImageStream

To ensure that phagocytosis measured by flow cytometry resulted in actual bead uptake, and not bead attachment alone, we performed imaging flow cytometry to assess the amount of bead attachment and internalization. Immune complexes were formed with HIVIG, IVIG and no antibody control as described above. WBCs were incubated with immune complexes at 4 °C and 37 °C for 1 h. Cells from 12 wells were then pooled, washed and stained with CD66b-AF647 (Biolegend) and DAPI (Invitrogen). Bead attachment and internalization by CD66b^+^ cells were visualized using an ImageStreamX MkII (EMD Millipore) across 500,000 independent neutrophil events (60× objective, 405, 488 and 642 nm lasers, with extended depth of focus) and quantified using IDEAS 6 software utilizing the internalization module. An internalization feature was employed describing the ratio of fluorescence intensity of the beads inside the cell against whole cell intensity, with positive scores representing greater internalization.

### 2.7 Analysis of cell supernatants for neutrophil activity beyond phagocytosis

To analyze myeloperoxidase (MPO), lactoferrin, matrix metallopeptidase 9 (MMP-9) and cytokine release upon neutrophil stimulation with immune complexes, supernatants were collected from ADNP assays performed with WBCs, in which the cells were incubated with the immune complexes formed by HIV^−^ or HIV^+^ sera for 1 or 4 h. MPO and lactoferrin release were measured at a 1:10 dilution of the ADNP supernatant using ELISA kits (eBiosciences, Abcam) according to manufacturer's directions. MMP-9 secretion of undiluted samples was measured in an ELISA using a matched antibody pair kit (Abcam), a modified version of the manufacturer's protocol adapted for 384-well plates, and 1-Step Ultra TMB (Thermo Scientific). The presence of cytokines in undiluted supernatant was detected using a customized multiplex luminex bead panel (Millipore Sigma) according to the manufacturer's instructions.

## 3 Results

### 3.1 A flow cytometry-based neutrophil phagocytic assay

With accumulating interest in the potential role of antibodies in directing neutrophil activity in HIV-1 research and beyond, sample-sparing, highly flexible antibody-dependent neutrophil assays are urgently needed across fields. The ADNP assay described in this paper has been used successfully for the investigation of the humoral immune response during tuberculosis infection (Lu et al., 2016) and the analysis of HIV-1 and Ebola virus vaccine studies (Holtsberg et al., 2016; Sips et al., 2016; Ackerman et al., 2016) and represents a critical piece of the evolving systems serology platform (Ackerman et al., 2017). Given the heterogeneity of primary neutrophils (Garley and Jabłońska, 2018) as well as the fact that current neutrophil cell lines incompletely capture natural neutrophil functional diversity (Garley and Jabłońska, 2018), here we describe an in-depth optimized primary cell-based ADNP assay as well as experimental opportunities to expand the assay using controllable fixed-target antigen-coated beads.

**Fig. 1:**
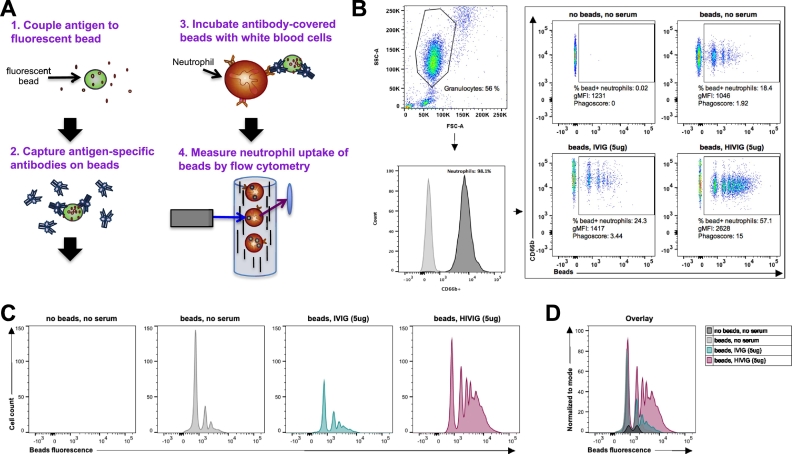
Schematic representation and gating strategy of the ADNP (antibody-dependent neutrophil phagocytosis) assay. (A) Fluorescent NeutrAvidin beads were coated with biotinylated human immunodeficiency virus 1 (HIV-1) YU2 gp120 and immune complexes were formed with purified antibody or serum. White blood cells (WBCs) were collected by ammonium-chloride-potassium (ACK) lysis from human blood using acid citrate dextrose (ACD) as a coagulant. WBCs were stimulated with immune complexes, stained for CD66b and fixed. Cell fluorescence was measured by flow cytometry and data were analyzed using FlowJo. (B) Granulocytes were gated based on SSC-A and FSC-A and the neutrophil subpopulation was determined using CD66b-PacBlue as a positive marker. Uptake of fluorescent immune complexes was quantified by measuring the percentage and gMFI (geometric mean fluorescence intensity) of FITC+ cells within the neutrophil population. Phagocytosis expressed as a “phagoscore” was calculated as described in the Materials and Methods. (C, D) Depiction of the data presented in B for the bead^+^ neutrophil population as histograms (C) and an overlay histogram (D).

Specifically, the ADNP assay can be divided into 2steps: i) the formation of immune complexes between antigen-specific antibodies and antigens, and ii) the phagocytosis of these immune complexes by neutrophils upon Fc-receptor (FcR)-binding on the cell surface. To quantify immune complex uptake, antigens are coated on fluorescent beads, allowing for the visualization of bead internalization by flow cytometry (Fig. 1a). First, the antigens of interest are biotinylated; biotinylated antigens are incubated with fluorescent NeutrAvidin coated beads, resulting in antigen capture. Excess antigen is eliminated, and beads are incubated with purified antibodies or serum to capture antigen-specific antibodies. For each assay, antigen-reactive and non-reactive controls are required to assess background and capture assay performance metrics. For example in this manuscript, PBS, serum samples and purified pooled Ig from HIV positive (HIVIG) or negative subjects (IVIG) were used as controls in assays using HIV-1 gp120 as a target antigen. After the incubation of the coupled beads with samples or controls, excess and non-specific antibodies are washed away, and the immune complexes are incubated with primary neutrophils. Supernatants are collected for secondary assays. Finally, bead internalization is quantified in live neutrophils by flow cytometry (Fig. 1B-D). A phagoscore is calculated to express both the number of neutrophils that have taken up beads, as well as the number of beads taken up by distinct cells. To develop this assay, several steps were optimized, described below.

### 3.2 Fractionated and un-fractionated neutrophils drive antigen-specific ADNP

In vivo, neutrophils function in the presence of other immune cells, and thus, the source of blood as well as the milieu in which neutrophil phagocytosis is assessed might impact the outcome of the ADNP assay. First, we assessed the importance of other WBCs for neutrophil function in the ADNP assay, since T cells and NK cells have been shown to promote neutrophil survival and phagocytosis (Bhatnagar et al., 2010; Pelletier et al., 2010; Agrati et al., 2009).

**Fig. 2:**
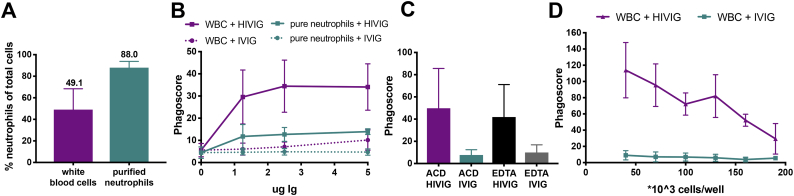
Effect of neutrophil source and cell number on phagocytosis. (A) After purification, CD66b^+^ neutrophils were gated. (B-D) ADNP assays were performed using HIVIG (pooled Ig from HIV-1^+^ subjects serving as a positive control), IVIG (pooled Ig from HIV^−^ subjects serving as a negative control) and HIV-1 gp120 to assess the phagocytic ability of neutrophils present in WBCs or purified neutrophils (B). The graphs depicts the influence of the anti-coagulants, ACD or ethylenediaminetetraacetic acid (EDTA), on WBCs or purified neutrophils (C), or the impact of WBC number for phagocytosis (D). For all presented assay results, phagoscores are displayed as mean ± SD of at least two independent experiments.

We compared ADNP induced by purified neutrophils (88.0% of population neutrophils) and neutrophils present in total WBCs (49.1% neutrophils) (Fig. 2A) using HIV-1 gp120 coated beads in the presence of the positive control IgG pool, HIVIG, or the negative IgG pool, IVIG. Neutrophils are captured based on their expression of CD66b. Our results indicate that both WBC and purified neutrophils are able to take up immune complexes; however, phagocytosis by WBCs provided a better signal-to-noise ratio (3.4–4.9 (WBC), 2.5–2.9 (purified neutrophils)) and higher phagoscores at equal cell numbers, potentially due to either the density of neutrophils or the presence of other immune cells able to promote enhanced phagocytosis (Fig. 2B). Thus, to develop the enhanced signal-based assay and mimic functions that may be observed in a whole blood system, a WBC-based assay was selected for further development. Conversely, while the magnitude of phagocytosis was lower, pure neutrophil populations can also be used as a source of cells to profile antigen-specific ADNP activity, particularly if downstream neutrophil-specific readouts are desired.

### 3.3 Anti-coagulant and cell density modulate neutrophil phagocytosis

Given the fact that anti-coagulants have been shown to influence neutrophil activation and lactoferrin secretion (Freitas et al., 2008; Engstad et al., 1997), we evaluated the impact of blood anti-coagulants on neutrophil phagocytosis. No significant difference was observed between the phagocytic activity of non-enriched neutrophils derived from ACD or EDTA anti-coagulated blood (Fig. 2C). However, because ACD blood gave higher fold over background phagoscores, ACD blood was used moving forward.

Finally, to address the impact of cell number on ADNP, we measured phagocytosis induced by HIVIG and IVIG using different densities of WBCs (Fig. 2D). Increased cell numbers decreased the efficiency of neutrophil phagocytosis, pointing to the importance of using an optimal effector to target (E:T) ratio to successfully measure phagocytosis. Overall our results demonstrate that non-enriched neutrophils derived from ACD blood, seeded at a cell density of between 40,000–70,000 cells/well are able to drive high level phagocytosis over a range of antibody concentrations. In the light of these results, we incorporated 50,000 cells/well as the standard in our protocol.

### 3.4 The production of immune complexes for neutrophil phagocytosis assays is fast and requires low amounts of antigen

To minimize the required sample input, the ADNP assay was optimized to not only work with purified Ig but also plasma samples. Since antigen-specific titers differ in plasma, the identification of an optimal dilution is critical to ensure sensitivity and resolution in the ADNP assay.

**Fig. 3:**
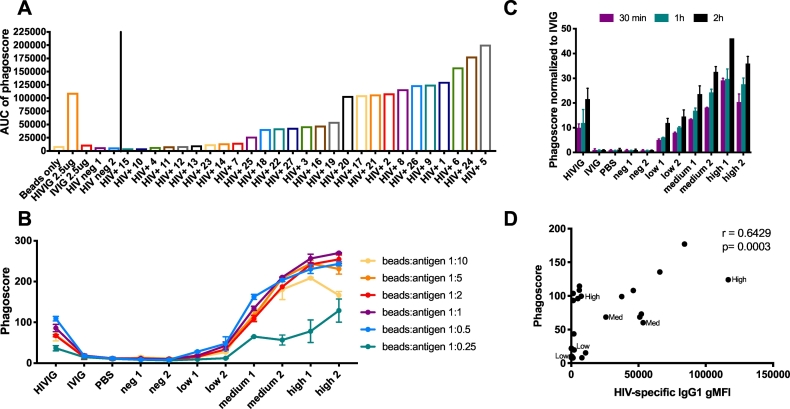
Optimization of antigen input and incubation times for the formation of immune complexes. (A) Sera from 27 HIV^+^, and 2 HIV^−^ patients were used to obtain a range of background, low, medium and high phagocytic responses using HIV-1 gp120 as an antigen. No antibody, IVIG and HIV^−^ serum were used as a negative control and HIVIG was used as a positive control. Phagoscores were measured and area under the curve values of 1:10, 1:100 and 1:1000 diluted samples were calculated. We chose donor 4 and 23 to represent low, donor 16 and 19 to represent medium and donor 5 and 24 to represent high phagocytic activity and these were used in the following experiments. The impact of (B) antigen amounts and (C) incubation time on phagocytosis was assessed. Results are shown as the mean ± SD phagoscore from two independent experiments. (D) The correlation plots depicts the non-parametric Spearman correlation of IgG1 titers and ADNP reactivity aginst HIV-1 gp120-specific antibodies in the tested serum samples. The samples selected for high, medium and low activity are indicated.

First, we performed an HIV-1 gp120-specific ADNP assay using 27 serially diluted sera from HIV-1^+^ individuals (Fig. 3A) to obtain a range of samples that could be used for further optimization. Based on the phagoscores obtained from each sample at different dilutions, we calculated the area under the curve (AUC), providing a measure of phagocytic activity over a dilution series instead of a single sample dilution. We identified 2 samples each with low (donor 4 and 23), medium (donor 16 and 19) and high (donor 5 and 24) HIV-1 gp120-specific phagocytic activity and these 6 samples were used for all future optimization assays to represent the full breadth of phagocytic activity present in the sample set.

To next determine the optimal antigen to bead ratio (Fig. 3B), the ADNP assay was conducted using beads coupled with 0.25, 0.5, 1, 2, 5 or 10 μg HIV-1 gp120 per μl of beads. The data show that 0.5 μg of HIV-1 gp120 per μl beads is sufficient for coating. To ensure robust results throughout all experiments a slightly higher concentration of 1 μg of antigen per 1 μl beads was selected for all further experiments resulting in a theoretical ratio of 7.5 × 10^5^ molecules of HIV-1 gp120 per bead. Importantly, optimal coating of beads may differ across antigens of different sizes and will require similar optimization.

Finally, we examined the optimal amount of time for the generation of the immune complexes (Fig. 3C). An ADNP assay was performed in which the selected HIV-1^+^ samples were incubated with the beads for different lengths of time. Immune complexes were formed in as little as 30 min, and differences between the samples with low, medium and high phagocytic activity were analyzed. Longer incubation times improved the signal-to-noise ratio observed between the samples, suggesting a 2 h incubation is optimal for the measurement of ADNP activity. HIV-specific IgG titers correlate with ADNP (Fig. 3D). However, a subset of low titer-samples exhibited medium ADNP activity and high ADNP activity was seen from a sample with 15-fold less HIV-specific titers, indicating titer independent activity and suggesting that measuring titers is not sufficient to predict ADNP activity, particularly at mid-to-low titers (Fig. 3D).

### 3.5 Immune complex uptake by neutrophils is fast but further improves with longer incubation periods

**Fig. 4:**
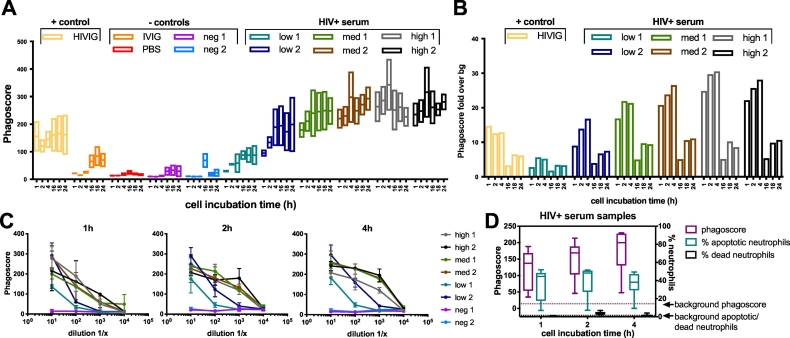
The influence of incubation time on phagocytosis and cellular quality. (A) HIV-1 gp120 immune complexes generated using a 1:100 serum dilution were incubated with neutrophils for 1, 2, 4, 16, 18 and 24 h. The mean, minimum and maximum values are displayed. (B) The same data presented in (A) is expressed as fold over background over the HIV^−^ serum sample in the bar graph. (C) The same assay as in (A) was performed with titrated samples and performed at incubation times of 1, 2 or 4 h. The mean ± SD phagoscore is displayed. (D) Neutrophil quality after a 1-4 h stimulation with HIV-1 gp120 immune complexes in an ADNP assay was assessed by labeling the cells with live/dead stain and Annexin V in addition to the standard staining. The median phagoscore or percent apoptotic or dead cells are shown.

To identify the optimal amount of time for efficient immune complex uptake by neutrophils in the ADNP assay, we performed an experiment probing changes in ADNP following 1, 2, 4, 16, 18 or 24 h of incubation of immune complexes with neutrophils (Fig. 4A). The 1 h time point was sufficient to measure antibody mediated neutrophil phagocytosis over a wide range of samples and responses; however, the signal-to-noise ratio calculated between HIV^+^ and HIV^-^ samples improved over time reaching its peak after 4 h of incubation (Fig. 4B). Beyond the 4 h timepoint, background levels increased, resulting in lower phagoscore over background (Fig. 4B), suggesting that 4 h is optimal for the interrogation of specific ADNP.

To exclude any potential biases in ADNP readouts due to serum dilutions, we next examined the influence of sample dilutions on 1-4 h incubation times (Fig. 4C). At all incubation times the best resolution between samples was observed at a 1:100 dilution, with samples with low phagocytic activity still exhibiting detectable responses, while medium and high ADNP-mediating samples could still be differentiated. Thus, a 1:100 dilution was used for further experiments. Notably, the reactivity of one sample (“high 1”) did change with the incubation time, exhibited high reactivity at 1 h and only moderate reactivity after a 4 h incubation. We hypothesize that this phenomena is due to neutrophil competitive activity, where neutrophils likely drive rapid early clearance of complexes that is then attenuated to a certain extent to prevent over-active inflammatory or immune activation. Thus, the ADNP assay can be conducted using 1-4 h incubation time, to capture different antibody subpopulations capturing different neutrophil recruiting activities. Given our particular interest in interrogating the rapid response of neutrophils, occurring immediately upon immune complex detection, we selected a 1 h incubation time point for further assay development.

Finally, because neutrophils have a short half-life of 4.3–17.5 h (Dresch et al., 1971; al-Janabi et al., 1993), ADNP results generated using longer incubation times might be impacted by a decline in neutrophil quality. Thus, the quality of neutrophils was assessed over multiple ADNP incubation times (Fig. 4D). Given the short half-lives of neutrophils, neutrophils were stained with Annexin V to probe the onset of apoptosis of the cells as well as a live/dead marker. No difference in the amount of apoptotic or dead neutrophils was observed between the 1, 2 or 4 h incubation period (mean % of apoptotic neutrophils with SD: 36.0 ± 17.8 (1 h), 40.1 ± 17.3 (2 h), 36.2 ± 14.2 (4 h); mean % of dead neutrophils with SD: 0.7 ± 0.3 (1 h), 3.8 ± 1.8 (2 h), 1.7 ± 1.2 (4 h)) indicating that neutrophil viability is not a concern in the ADNP assay with incubation times for the phagocytosis of immune complexes of up to 4 h.

### 3.6 The ADNP assay performs robustly and measures FcR mediated uptake of immune complexes

**Fig. 5:**
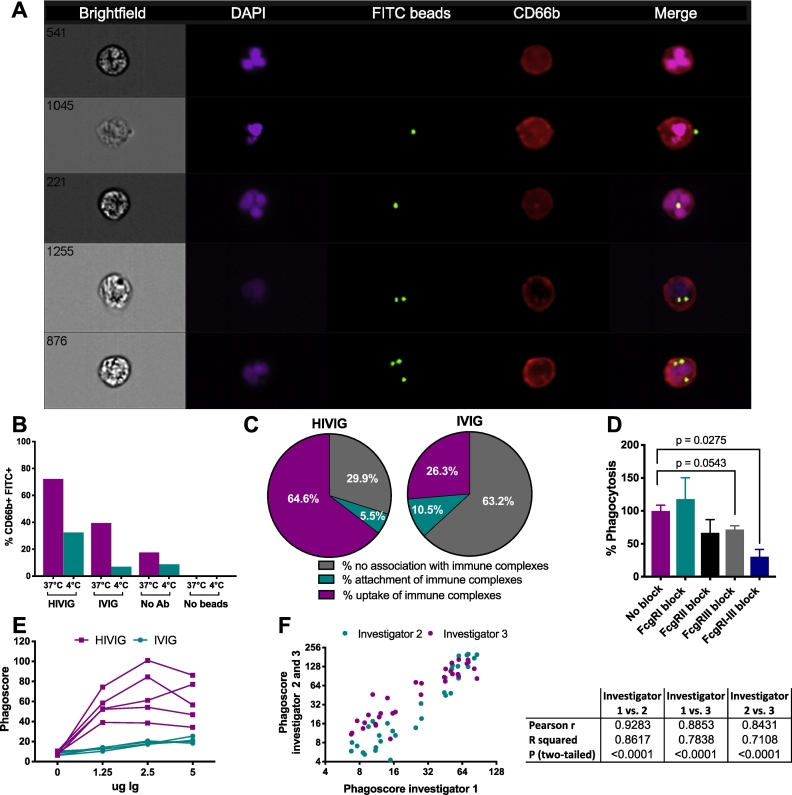
Characterization of neutrophil-immune complex interactions in the ADNP assay and assay reproducibility. (A-C) Neutrophil phagocytic uptake or attachment of HIV-1 gp120-immune complexes to neutrophils was measured by imaging flow cytometry after 1 h of incubation at 4 °C or 37 °C. Neutrophils were labeled with anti-CD66b (red), nuclei with DAPI (purple) and immune complexes were measured in the FITC channel (green). A total of 1000 cells from 500,000 pooled replicate wells from a single donor were analyzed. (A) Representative images of neutrophils are shown in the following order from top to bottom: no bead internalization, surface bead attachment, single bead internalization, and internalization of two or three beads by neutrophils. (B) The total percentage of neutrophils associated with fluorescent beads was calculated for the different conditions tested. (C) The data obtained for HIVIG and IVIG after an incubation of the immune complexes with the neutrophils at 37 °C is further analyzed to show the % of attached immune complexes as well as the % of phagocytosed immune complexes within the bead-associated neutrophil population. (D) Whether or not immune complex uptake was mediated by Fc gamma receptors (FcgRs) was assessed by performing an ADNP assay using HIVIG and IVIG with HIV-1 gp120-coated beads, in which single or all FcRs were blocked. Four independent experiments were performed. Displayed is the mean ± SEM phagoscore of HIVIG-containing samples normalized to the results in the absence of FcgR block. *P* values were calculated using a paired *T*-Test. (E) Donor-to-donor variability of neutrophils was assessed by conducting an ADNP assay using 5 different WBC donors and HIV-1 gp120 coated beads. (F) The robustness of the ADNP assay was assessed across different users. For this purpose, 3 different experimenters performed the same ADNP assay using HIV-1 gp120-coated beads, HIV-1^+^ samples, controls, and the same cell donors. For E-F, the results of a single experiment are shown. (For interpretation of the references to color in this figure legend, the reader is referred to the web version of this article.)

To ensure that the fluorescent signal measured in the ADNP assay by flow cytometry does indeed quantify immune complex phagocytosis, and not merely the binding of neutrophils to immune complexes, ImageStream was used to quantify % internalization (uptake) vs. % attachment (Fig. 5A). Neutrophils were incubated with immune complexes consisting of HIV-1 gp120 coupled to NeutrAvidin beads and HIVIG or IVIG at 37 °C as well as 4 °C. Neutrophils were identified using CD66b^+^ (red), and immune complexes were measured using FITC beads (green). Different states of bead association with neutrophils were clearly observed using this method (37 °C), including bead attachment and internalization (Fig. 5A). As expected, immune complexes formed using HIVIG at 37 °C resulted in 72.2% FITC^+^CD66b^+^ cells (uptake). In comparison, only 32.4% of CD66b^+^ cells were FITC^+^ at 4 °C. Additionally, only 39.5% or 6.9% neutrophils were FITC^+^ in the presence of control IVIG antibodies at 37 °C and 4 °C (Fig. 5B, respectively) suggesting most antigen-coated bead uptake was seen in the presence of HIVIG as quantified in Fig. 5C, left. In contrast, FITC-positivity was mostly external to both the nucleus and plasma membrane in the presence of IVIG (Fig. 5C, right). These imaging results support the finding that our flow-based ADNP assay accurately measures immune complex uptake, and that our readout is sufficient to resolve differences between samples.

To confirm that our assay was a measure of antibody-Fc mediated function, receptor-blocking experiments were also performed (Fig. 5D). As anticipated, blocking of FcγRII, III or all FcγRs resulted in a decrease of phagocytosis by 33.3%, 28.3% and 69.6%, respectively, suggesting the mechanism of bead internalization was directed through interaction of antibody-Fc with Fc receptors, likely largely driven by FcγR2 and 3, and was not non-specific.

Differences in the neutrophil donors did impact the overall phagoscore, but was always concordant. While the level of phagocytic reactivity of HIVIG against HIV-1 gp120 was variable across 5 blood donors, the assay background measured using IVIG remained stable (Fig. 5E). Thus, donor-to-donor variability provides the same resolution in qualitative differences in antibody function across plasma samples and donor-to-donor heterogeneity can result in differences in signal intensity, pointing to opportunities to mine for the impact of donor-dependent neutrophil functionality in the future.

Finally, to ultimately investigate whether the ADNP assay performs similarly between different operators, a key element for robust assay reproducibility, the assay performance was compared across three different users exploiting the same cell donor (Fig. 5F). Highly correlated results were observed between different experimenters. In summary, these findings suggest that the ADNP assay interrogates Fc-receptor mediated bead uptake that can be utilized to reliably investigate the role of neutrophil phagocytosis in a robust manner.

### 3.7 The ADNP assay is a broadly applicable tool for the investigation of neutrophil function

**Fig. 6:**
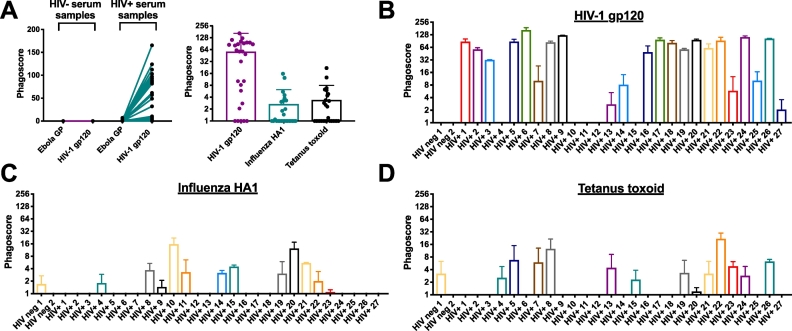
Neutrophil phagocytosis is antigen-specific. (A) ADNP assay specificity was analyzed by comparing the phagocytosis of immune complexes formed using HIV-1^+^ or HIV-1^−^ samples with HIV-1 gp120-, influenza HA1- or Ebola virus GP-antigen coated beads. (B-D) The results obtained in (A) show the phagocytic profiles obtained for each serum donor for HIV-1 gp120 (B), influenza HA1 (C) or tetanus toxoid (D). Two independent experiments were performed and the mean ± SD phagoscore is displayed without (A) or after (B-D) subtraction of the background (no antibody).

Neutrophils interact with a wide range of bacteria, fungi, parasites and viruses (Kobayashi et al., 2017). To understand whether the ADNP assay can be broadly applied to investigate neutrophil activity against other pathogens, the ADNP assay was performed using HIV-1^+^ and HIV-1^−^ sera against HIV-1, influenza virus, *Clostridum tetani* and Ebola virus antigens (Fig. 6A). As expected, none of the sera mediated phagocytosis against Ebola virus GP, which served as a negative control, as the donors were unlikely to have been exposed to the virus or the vaccine.

In contrast, a wide range of signals were observed for HIV-1 gp120 across HIV^+^ subjects, but not in HIV^-^ subjects, confirming that the ADNP assay detects antigen-specific signals (Fig. 6A, left panel). Similarly, heterogeneous influenza HA1- and tetanus toxoid-specific responses were observed (mean phagoscores 53 (HIV), 2.7 (influenza) and 3.4(tetanus); Fig. 6A, right panel). Critically, gp120-, HA1-, and tetanus-specific responses showed expected distinct profiles (Fig. 6B-D), highlighting the versatility of the assay and simple adaptation for additional antigen-specific antibody profiling.

### 3.8 The ADNP assay offers a unique opportunity to further probe down-stream antibody-induced neutrophil functions

**Fig. 7:**
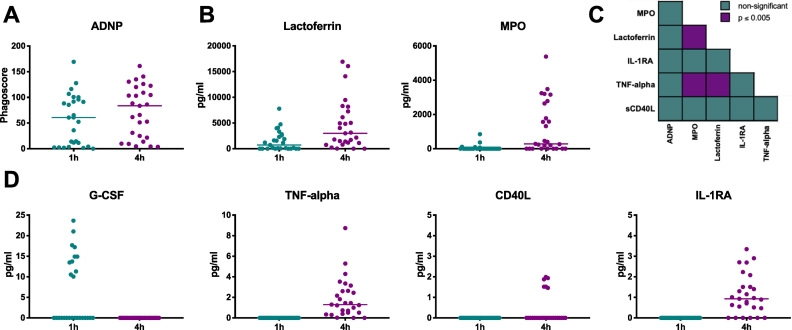
Secondary ADNP assays provide additional insights into antibody-mediated neutrophil activation. (A) Secondary readouts were performed on supernatants collected following incubation of 27 HIV^+^ sera as well as 2 HIV^−^ sera and HIV-1 gp120-coated beads with neutrophils at 1 or 4 h. (B) Neutrophil degranulation in the supernatant was assessed using human lactoferrin and myeloperoxidase (MPO) ELISAs. (D) Cytokine secretion into the supernatants was analyzed by luminex. Results are displayed for granulocyte-colony stimulating factor (G-CSF), tumor necrosis factor alpha (TNF-alpha), CD40 ligand (CD40L) and interleukin-1 receptor antagonist protein (IL-1RA). Figs. A, B and D show the averaged results of two independent experiments after subtraction of the background (HIV^−^ samples) with lines representing the median cytokine concentrations. (C) Non-parametric Spearman correlation of the results from A, B, and D.

Beyond phagocytosis, antibodies may deploy a broader range of neutrophil functions including degranulation and cytokine release to provide protection against foreign pathogens. Ideally, the in-depth analysis of other neutrophil functions beyond ADNP can be captured at no additional sample expense and with minimal effort. Specifically, to develop a set of robust secondary assays, supernatants were analyzed for secondary neutrophil functional readouts (cytokines and degranulation markers) after 1 or 4 h of incubation of neutrophils with HIV^+^ and HIV^−^ samples (Fig. 7).

As observed previously (Fig. 3A), the HIV-1^+^ serum samples showed a wide range of ADNP, which improved over longer incubation periods (Fig. 4C and Fig. 7A). Secondary markers of neutrophil functionality including MPO and lactoferrin were only detectable at the 4 h time point (Fig. 7B) and not at 1 h. MMP-9 was not detectable at either time point (data not shown). Additionally, a luminex assay was also used to further interrogate cytokine secretion in supernatants (Fig. 7D). Granulocyte-colony stimulating factor (G-CSF) secretion was exclusively measurable after 1 h of neutrophil incubation. In contrast, tumor necrosis factor alpha (TNF-alpha), CD40 ligand (CD40L) and interleukin-1 receptor antagonist protein (IL-1RA) signals were detectable after 4 h of immune complex stimulation. Conversely, Granulocyte/Macrophage Colony Stimulating Factor (GM-CSF) as well as interleukin 10, 1A, 1B and 6 were undetectable (data not shown). Next, we investigated whether the downstream neutrophil functions tracked with one another (Fig. 7C). Interestingly, in this sample set, a positive correlation was observed between TNF-alpha secretion and the release of MPO and lactoferrin containing granules, pointing to a potentially interesting neutrophil functional axis leveraged by HIV-1 gp120-specific immune complexes. Thus, this platform approach offers a unique opportunity to define whether similar or distinct axes may be utilized by other humoral immune responses. In conclusion, the analysis of degranulation markers and cytokines demonstrated that the ADNP assay could easily be combined with down-stream assays measuring lactoferrin, MPO or cytokine secretion without additional sample investment. Further, correlation analysis of the detected responses is a tool to unravel potentially new and meaningful relationships between functions present in neutrophil reactive profiles to specific pathogens or diseases.

## 4 Discussion

Non-neutralizing antibodies have been documented in protection against CMV (Nelson et al., 2018), Ebola virus (Holtsberg et al., 2016; Zeitlin et al., 2011), and influenza virus (DiLillo et al., 2014). Additionally, non-neutralizing antibodies were identified as correlates of protection against HIV-1 infection in the first moderately protective human vaccine trial (Rerks-Ngarm et al., 2009). Non-neutralizing antibodies have also been linked to protection in non-human primate studies using SIV (Barouch et al., 2015) and SHIV (Barouch et al., 2013), and recent data have specifically implicated ADNP as a correlate of immunity (Ackerman et al., 2018). Since neutrophils are among the most numerous cells in the blood, and are recruited rapidly into inflamed tissues, assays capable of dissecting their contribution to antibody-driven immunity may offer novel insights for the design and evaluation of vaccines. Thus, we developed a high-throughput, robust and extremely versatile assay to measure ADNP using antigen-coated fluorescent beads.

Though other neutrophil mediated assays have been previously described, the assay presented here uses primary neutrophils, is sample sparing, antigenically flexible, high-throughput, and extendable to many secondary analyses. For instance, by using primary neutrophils instead of a cell line the unique opportunity to broadly capture donor-to-donor variability and natural neutrophil reactivity to immune complexes, which may be key to defining correlates of immunity and/or novel mechanisms of antibody action, can be explored. Prior experimental design efforts speculated that the use of primary neutrophils would result in highly heterogeneous results that would likely be uninformative, however, here we observed robust concordance across donors (Fig. 5E), albeit with different magnitudes. This striking concordance highlight the possibility that this assay may also enable the interrogation of patient-derived neutrophils across populations and/or disease states to gain insights on the functional consequences of changes in these innate effectors, and may provide an opportunity to interrogate ADNP in a population-specific manner, potentially key to dissecting correlates of immunity or customizing therapeutics for a particular immunologic condition.

Low background phagocytic uptake in the absence of antibodies point to the highly specific nature of this primary neutrophil assay that may be effectively and easily deployed to interrogate functions across a broad range of target antigens. Thus, this platform may even be deployed more broadly to define neutrophil dysfunctional biomarkers that may precede neutropenia across infectious, oncological, and even transplantation settings, and define novel drugable axes to enhance neutrophil mediated antibody-immunity in non-infectious disease states. Furthermore, the assay may also be deployed to more broadly explore neutrophil functional variability related to genomic perturbations in FcRs. For example, variability may be linked to single nucleotide polymorphisms (SNPs) and variation in the copy number of FcgR encoding genes which impact the expression level, affinity and functionality of FcRs (Bournazos et al., 2009). Finally, given that environmental factors, including inflammation, can shape the functional profile of neutrophils due to their high level of plasticity (Tsuda et al., 2004; Chakravarti et al., 2009) and heterogenic composition within patients (Garley and Jabłońska, 2018; Hellebrekers et al., 2017), the use of primary neutrophils may be vital to fully explore the mechanistic role of neutrophils in protection or pathology across diseases. Thus, cell lines fail to comprehensively recapitulate the diversity of neutrophil populations, functions, and responsiveness, thereby rendering primary ADNP assays a robust and versatile solution in profiling neutrophil activity.

Beyond neutrophil phagocytic clearance, neutrophils may contribute to anti-pathogen immunity via the release of immune-activating factors as well as via the release of NETs (Rosales et al., 2017). Differences in lactoferrin, MPO and cytokine secretion were detectable in supernatants, highlighting the flexibility of downstream secondary assays. In addition, other neutrophil functions like the secretion of leukotrienes and reactive oxygen species as well as the release of neutrophil elastase, a measure for NET formation, can also all be potentially assessed using the supernatants from the ADNP assay. Therefore, the ADNP assay enables the generation of detailed functional profiles of neutrophils without additional sample input, which can extensively exceed the 7 parameters shown here (Fig. 7).

Additionally, while assays using native pathogens or infected cells have been developed (Dri et al., 2002; Rodríguez et al., 2001; Richard et al., 2018), these targets may present many target antigens as well as different states of the antigens, rendering it difficult to interpret the specific functional activity of particular antigen-specific antibody populations. Conversely, the bead-based system offers a simple tractable means to capture antibody functionality against single, well-defined antigens of interest in a more controllable manner. Several strategies can be used to couple the antigens to the beads including enzymatic biotinylation, direct covalent coupling to beads, or even engineered tags that may help orient the target molecule on the bead. Importantly, it is critical to ensure that the coupling strategy maintains the antigenic character of the target antigen, using recombinant monoclonals or known reactive/non-reactive sera, to ensure that the maintenance of antigenicity.

In summary, the ADNP platform described in this article provides a tool for the detailed characterization of neutrophil functions using minimal amounts of sample. Thus, the ADNP platform can be used to support the global effort of identifying novel correlates of protection for diseases for which no known correlate of immunity exists, to interrogate plasma antibody functionality across infectious and non-infectious diseases, and even dissect antibody mechanism(s) of action.

## Declarations of interest

None.
